# Anomaly Detection via Progressive Reconstruction and Hierarchical Feature Fusion

**DOI:** 10.3390/s23218750

**Published:** 2023-10-27

**Authors:** Fei Liu, Xiaoming Zhu, Pingfa Feng, Long Zeng

**Affiliations:** Tsinghua Shenzhen International Graduate School, Tsinghua University, Shenzhen 518055, China; buaaliufei@163.com (F.L.); ZxiaomingThu@163.com (X.Z.); feng.pingfa@sz.tsinghua.edu.cn (P.F.)

**Keywords:** anomaly detection, progressive reconstruction, hierarchical feature fusion, attention mechanism, packaging chips

## Abstract

The main challenges in reconstruction-based anomaly detection include the breakdown of the generalization gap due to improved fitting capabilities and the overfitting problem arising from simulated defects. To overcome this, we propose a new method called PRFF-AD, which utilizes progressive reconstruction and hierarchical feature fusion. It consists of a reconstructive sub-network and a discriminative sub-network. The former achieves anomaly-free reconstruction while maintaining nominal patterns, and the latter locates defects based on pre- and post-reconstruction information. Given defective samples, we find that adopting a progressive reconstruction approach leads to higher-quality reconstructions without compromising the assumption of a generalization gap. Meanwhile, to alleviate the network’s overfitting of synthetic defects and address the issue of reconstruction errors, we fuse hierarchical features as guidance for discriminating defects. Moreover, with the help of an attention mechanism, the network achieves higher classification and localization accuracy. In addition, we construct a large dataset for packaging chips, named GTanoIC, with 1750 real non-defective samples and 470 real defective samples, and we provide their pixel-level annotations. Evaluation results demonstrate that our method outperforms other reconstruction-based methods on two challenging datasets: MVTec AD and GTanoIC.

## 1. Introduction

**Figure 1:**
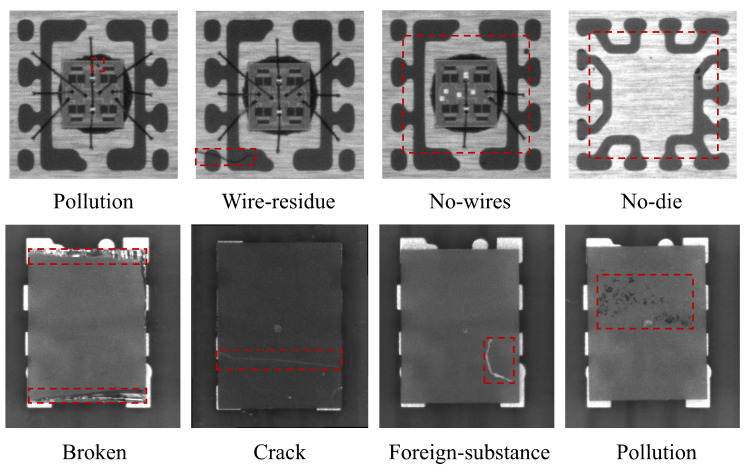
Several common types of chip surface defects. The defect area is highlighted with a dashed rectangle.

Deep-learning-based defect detection methods encounter challenges when applied in practical scenarios, as exemplified by the semiconductor industry. Firstly, manufacturing processes often produce defects infrequently, making it difficult to obtain a sufficiently large and varied dataset. Secondly, defects are often unevenly distributed among different products or batches, complicating the sampling process. Thirdly, the acquisition of annotated data can be a labor-intensive task. Furthermore, semiconductor devices use various models and undergo rapid iterations, which pose challenges for traditional supervised methods to keep pace. Additionally, in some cases, the lack of model interpretability may be deemed unacceptable. Figure 1 shows several common forms of chip defects.

To adapt to industrial scenarios, anomaly detection techniques have been extensively studied. Unlike traditional deep-learning-based detection methods that use CNNs to learn high-dimensional representations of defects from large-scale defect datasets, anomaly detection models are trained exclusively on normal samples. During inference, they possess the capability to distinguish whether input samples belong to the normal class or the anomaly class.

Reconstruction-based methods have achieved great progress in anomaly detection. These methods are trained with the assumption of a generalization gap, signifying that the model can successfully reconstruct normal samples but fails to do so with anomalies [1]. For example, reconstruction models based on autoencoders [2,3,4,5,6,7,8,9] or generative adversarial networks (GANs) [10,11,12] aim to reconstruct normal images and locate anomalies based on the reconstruction error. However, due to their powerful generalization ability, abnormal regions may still remain anomalous even after reconstruction [13]. To overcome this difficulty, previous works [14,15,16] treated anomaly detection as an inpainting task and used partial masking to reduce the possibility of defect reconstruction. However, they performed poorly on random-pattern-heavy classes such as tiles or metal nuts. A common drawback of the generative methods is that they only learn the model from anomaly-free data, and are not explicitly optimized for discriminative anomaly detection. Some recent attempts [12,17,18] introduced different anomaly simulation strategies to address this limitation. Among them, DRAEM [17] demonstrated excellent performance. Nevertheless, these methods struggled with overfitting to synthetic appearances, hindering their ability to generalize to real anomalies.

To summarize, the performance of reconstruction-based methods has long been limited by several tough problems: (1) Continuously enhancing the network’s fitting capabilities can result in a breakdown of the generalization gap. (2) When intentionally suppressing the network’s generalizability, the quality of reconstructed images deteriorates. (3) Unlike real defects, simulated defect patterns are often too pronounced, leading to network overfitting.

To overcome these limitations, we revisit DRAEM and further improve it in two aspects, based on progressive reconstruction and hierarchical feature fusion, naming it PRFF-AD. This method consists of a reconstructive sub-network and a discriminative sub-network. The former sub-network is responsible for learning anomaly-free reconstructions, while the latter sub-network combines the original image, reconstructed image, and intermediate feature information to generate a high-fidelity per-pixel anomaly detection map. Specifically, for the reconstructive sub-network, to improve the reconstruction quality without increasing the network’s fitting capacity, we incrementally return the previously reconstructed image back into the sub-network for further refinement. As for the discriminative sub-network, to address the optimization trade-off between classification and localization accuracy in U-Net [19], we employ Swin transformer [20] with an UperNet [21] architecture, which captures long-range dependency through an attention mechanism and increases the sensory field to improve classification accuracy while ensuring localization accuracy. In addition, to handle the introduction of new anomalies during the reconstruction process and to avoid overfitting to simulated defect regions, we provide not only the original and reconstructed images but also feature information from intermediate layers of reconstructive sub-networks as inputs to the discriminative sub-network, thereby enriching the network’s judgment information. To support our research, we construct a large dataset for packaging chips, named GTanoIC, with 1750 real non-defective samples and 470 real defective samples, and we provide their pixel-level annotations. To the best of our knowledge, this is currently the largest real surface defect dataset for chips. This dataset can be used for subsequent research and evaluation.

Experimental results demonstrate that our proposed method significantly enhances the performance of DRAEM. On the MVTec AD public dataset [22], it raises the image-level AUROC from 98.0% to 99.1% and pixel-level AUROC from 97.3% to 98.0%, surpassing other similar reconstruction-based methods. Moreover, it achieves state-of-the-art performance on the GTanoIC dataset, achieving an image-level AUROC of 97.5% and a pixel-level AUROC of 98.3%. On average, compared with the baseline, our method improves the image-level AUROC by 11.6% and the pixel-level AUROC by 6%.

In summary, the main contributions of this paper are as follows:

- We incorporate progressive reconstruction and feature fusion into DRAEM while enhancing its understanding of hierarchical features through technologies such as Swin transformer and UperNet. Our proposed method outperforms similar algorithms on both the MVTec-AD public dataset and GTanoIC chip dataset.
- We construct the largest real chip surface defect dataset to the best of our knowledge. It consists of 1750 real non-defective samples, 470 real defective samples, and pixel-level annotations.

## 2. Related Works

Methods for image reconstruction in anomaly detection, in contrast, include autoencoders [3,4,5,6], variational autoencoders [7,8,9], and generative adversarial networks (GANs) [10,11]. Most of them mainly input normal images and train the network to extract high-dimensional features and then reconstruct them into normal images. Since the input and output of the training process are equal, the network may have compression and decompression capabilities but not be able to learn semantics. Therefore, OCR-GAN [12] proposed to handle the sensory anomaly detection task from the perspective of frequency, since different frequency bands contain different types of semantic information. In contrast, we employ a discriminative sub-network consisting of a Swin transformer and UperNet to understand the differences between pre- and post-reconstruction. We supervise its learning of defect semantics through an anomaly simulation strategy. This approach enables automatic learning of an appropriate distance measure, yielding accurate segmentation maps.

In addition, neural networks’ robust learning capabilities can lead to accurate reconstruction of abnormal regions too. To alleviate this problem, methods such as SMAI [14], RIAD [15], and InTra [16] employed partial masking and reconstruction of images, achieving promising results. They disrupt the integrity and coherence of defects during detection, thereby reducing the possibility of being reconstructed. However, intentionally suppressing the reconstruction network’s generalizability can result in blurry, abstract reconstructions, and may introduce new “anomalies” during detection. Unlike these methods, we introduce progressive reconstruction to enhance the quality of reconstructed images without augmenting the network’s fitting capability.

Many recent self-supervised learning techniques have been introduced to explicitly learn the potential differences between normal and abnormal samples during training. Specifically, CutPaste [18] is dedicated to generating spatial irregularities through cut&paste augmentation as a rough approximation of real defects. However, the distribution of these simulated defects is far from the actual defect distribution. In contrast, DRAEM generates simulated defects through random noise and natural image transformations, and then abnormalizes normal images. However, simulated defects often have clear edges and distinct patterns, and this gap makes the network prone to overfitting during training and results in poor detection performances on real defect images during inference. More recently, EdgRec [12] proposed to reconstruct the simulated anomalous image from its gray value edge to minimize the chances of restoring anomalous areas. In our opinion, it is not appropriate to directly compare simulated defects with reconstructed images, as this can exacerbate the network’s overfitting problem. Therefore, we fuse images before and after reconstruction with feature maps from different stages of the reconstruction process, providing the discriminative sub-network with rich pixel-level and feature-level information.

## 3. GTanoIC Dataset

**Figure 2:**
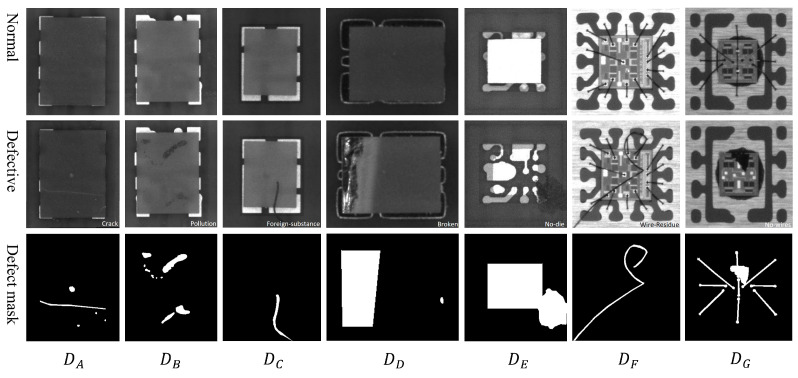
Seven types of chip in GTanoIC sub-dataset.

This paper constructed an anomaly detection dataset (named GTanoIC) consisting of seven types of chips, as shown in Figure 2. Five types are flip chips, while the other two types have chips with solder wires. Some minor defects have low contrast, making it difficult to perform defect detection using traditional threshold-based blob analysis methods. Detailed statistics on the inverted chips and solder joint chips are presented in Table 1 and Table 2, respectively.

For each type of chip, 200 normal samples were selected to comprise a training set, while the remaining samples were used as a test set. The test set includes not only normal images but also real chip defect images with common anomalies such as scratches, contamination, slight corner fractures, and misalignment. Furthermore, pixel-level annotations were provided for each test image to evaluate the algorithm’s anomaly localization performance.

## 4. Method

**Figure 3:**
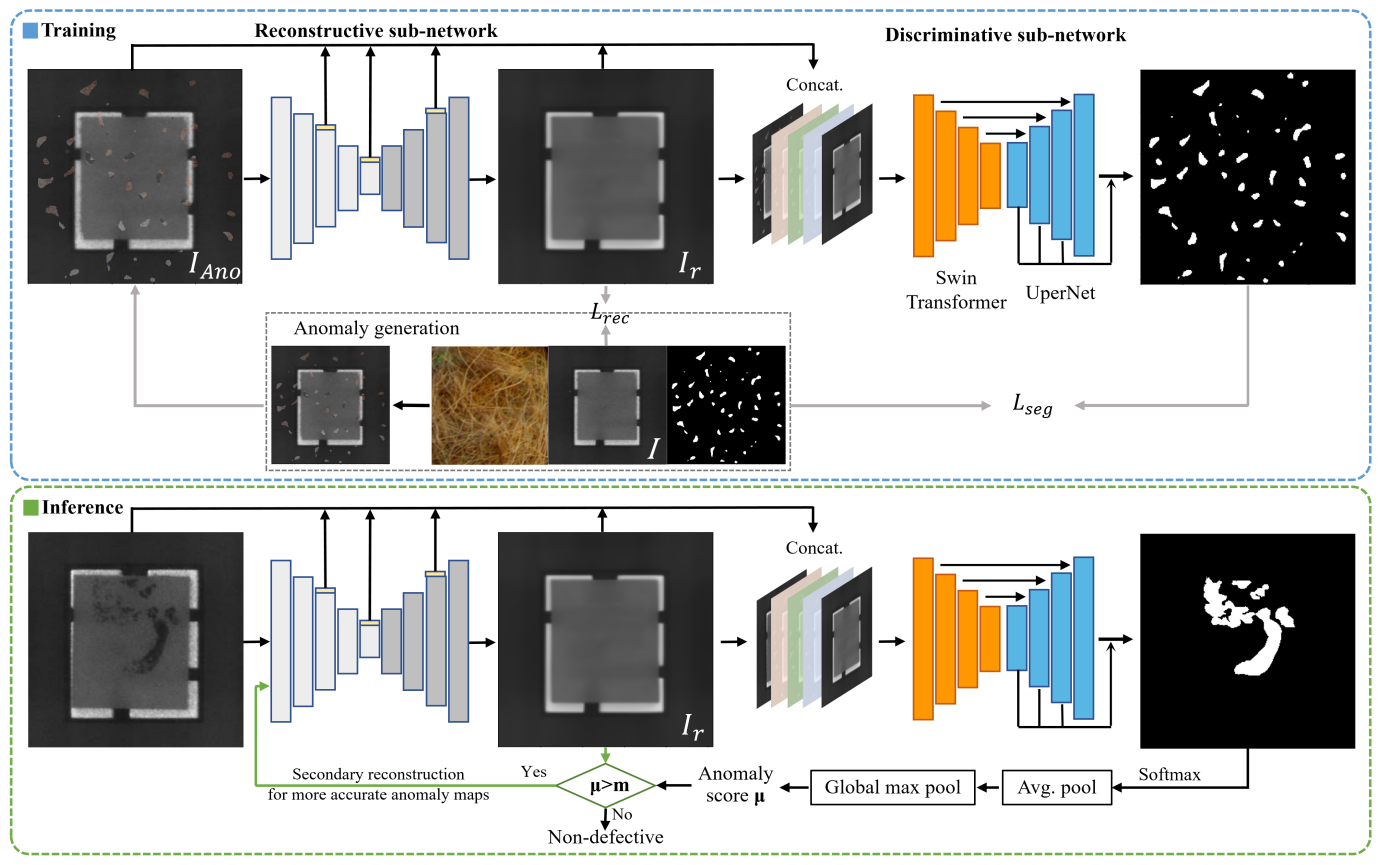
An overview of PRFF-AD. At training time, simulated anomalous samples are implicitly detected and repaired by the trained reconstructive sub-network. The output reconstructed image, its original image, and intermediate feature information are then fused and fed into the discriminative sub-network to generate an anomaly map. At inference time, images with a high anomaly score μ are reconstructed twice to obtain a more accurate detection result.

To address the problems with current reconstruction-based anomaly detection methods, we designed a novel network PRFF-AD, as shown in Figure 3. It consists of a reconstructive sub-network and a discriminative sub-network. The former is used to learn anomaly-free reconstructions, while the latter is designed to generate high-fidelity per-pixel anomaly detection maps. In this section, we describe the two sub-networks in detail.

### 4.1. Reconstructive Sub-Network

The goal of the reconstructive sub-network is to reconstruct anomalous images into normal ones. First, we generate simulated defects for training samples using the same anomaly simulation strategy as employed in DRAEM. In particular, we apply random augmentation to anomaly texture image *A*, subsequently mask it with a noise mask *M* (randomly sampled from Berlin noise), and then blend it with a non-defective sample *I* to generate a simulated anomalous image. Next, the simulated anomalous images are fed into the reconstructive sub-network. The encoder first transforms the images into high-dimensional feature representations, and then the decoder decodes them into images of the same size as the original input. During this training process, the network gains a semantic understanding of the images, thus enabling it to reconstruct anomalies into normal patterns.

At inference time, images with anomalies are repaired by the reconstructive sub-network to become defect-free images, which are then compared with the original images to identify anomalous regions. It is evident that the quality of reconstructed images directly affects anomaly detection. However, existing reconstructive networks face a contradiction during training: the network’s reconstruction ability improves continuously during training with normal samples, resulting in failure to maintain the generalization gap, resulting in some anomalous regions remaining as anomalies even after reconstruction; in this case, the images cannot be completely reconstructed into a normal image.

**Figure 4:**
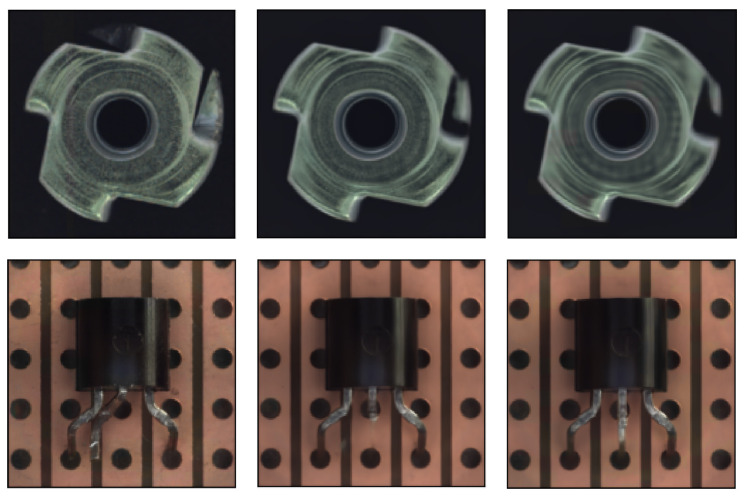
From left to right, the anomalous images are sequentially passed through the reconstructive sub-network (n = 0, 1, 2).

**Figure 5:**
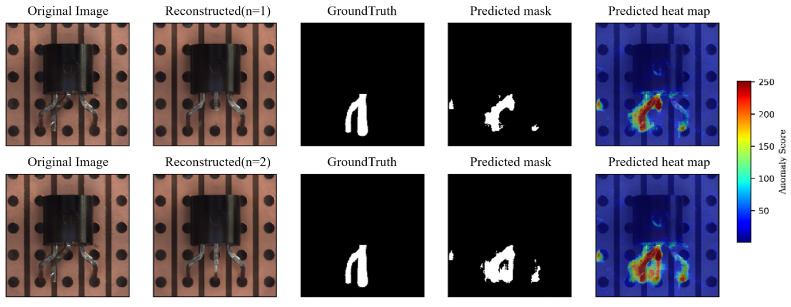
Further reconstruction (n = 2) provides a more accurate prediction mask and therefore improves pixel-level accuracy.

Therefore, to further improve the quality of the reconstructed image without increasing the network’s generalizability, we propose a progressive reconstruction approach. This process is displayed in Figure 3. We found that by feeding the reconstructed image back into the reconstruction sub-network for another round of reconstruction, we can obtain higher-quality reconstructed images. After each reconstruction, the abnormal regions in the image become smaller, and the patterns tend to normalize, thus providing more information for the next round of reconstruction and thereby enhancing the overall reconstruction effectiveness. The effect of progressive reconstruction is shown in Figure 4: from left to right, the anomalous images go through the reconstructive sub-network in turn (n = 0, 1, 2). In the metal nut case, the anomalous area shrinks as the number of reconstructions increases. In the transistor case, the missing and bent pins of the transistor are gradually repaired. In Figure 5, it can be observed that further reconstruction improves the pixel-level accuracy.

During inference, we smooth the output anomaly map by local average pooling and then compute its anomaly score μ by taking the maximum value of the smoothed anomaly map. It is worth mentioning that we perform progressive reconstruction only for samples with high anomaly scores (μ>m). *m* is a hyperparameter, which is taken as 0.5 in this paper. In industrial scenarios, the product yield rate is very high; hence, in practical application of this method, only a very small number of samples need to be reconstructed twice. In general, progressive reconstruction does not affect the overall detection speed.

The reconstructive sub-network is trained by the difference between the reconstructed image and its ground truth as a loss. We first use MSE loss to measure this difference, which is defined as follows:

(1) $$L_{MSE}\left(I_{r},I\right)=\frac{1}{m}\sum_{i=1}^{m}{\left(I_{r}-I\right)}^{2}.$$

However, MSE loss directly calculates the error between each pixel and ignores connections between pixels. Tested images often have rich local structure information, and MSE cannot effectively relate local structures. Therefore, similar to other reconstruction-based methods, the proposed method also incorporates SSIM loss. As shown in Equation (2), the structural information of the whole image is considered based on three comparative measures between the reconstructed image and normal image: luminance, contrast, and structure.

(2) $$ssim\left(I_{r},I\right)=f\left(l\left(I_{r},I\right),c\left(I_{r},I\right),s\left(I_{r},I\right)\right).$$

Here, $l(I_{r},I)$ is a luminance comparison function, which is estimated as the average gray scale of images. $c(I_{r},I)$ is a contrast comparison function estimated as the standard deviation of images. Finally, $s(I_{r},I)$ is a structural comparison function computed by dividing an image by its own standard deviation. Loss $L_{ssim}$ is calculated by Equation (3), which takes into account the differences in brightness, contrast, and pattern structure of images. *H* and *W* represent the height and width of the images, respectively.

(3) $$L_{ssim}\left(I_{r},I\right)=\frac{1}{H*W}\sum_{i=1}^{H}\sum_{j=1}^{W}1-ssim{\left(I_{r},I\right)}_{\left(i,j\right)}.$$

The complete reconstruction loss is therefore:

(4) $$L_{rec}\left(I_{r},I\right)=L_{MSE}\left(I_{r},I\right)+L_{ssim}\left(I_{r},I\right).$$

### 4.2. Discriminative Sub-Network

**Figure 6:**
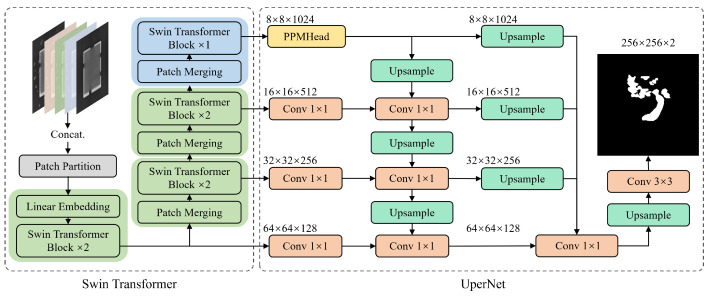
Structure of the discriminative sub-network.

Due to the lack of one-to-one correspondence between the reconstructed image and its original counterpart at the pixel level, pixel-by-pixel comparison-based anomaly discrimination methods [15,16] tend to result in a higher false detection rate. To mitigate this issue, the use of deep-learning-based discriminative networks can detect anomalous regions at both feature and semantic levels, thereby improving the accuracy of anomaly detection. Our proposed discriminative sub-network consists of Swin transformer and UperNet. The specific structure is shown in Figure 6. Multi-level input information is fed to Swin transformer to capture high-order features, and then UperNet performs up-sampling and summarizes the pixel-level difference information, ultimately outputting the anomaly score map.

Reconstruction-based anomaly detection methods commonly encounter two issues. First, compared with real defects, the simulated defect regions in images containing anomalies often have clear edges and significantly different patterns from normal regions. This situation makes simulated anomalies clearly stand out in the images, leading to potential overfitting problems during training of the discriminative sub-network. Second, the reconstruction process of the network may not perfectly restore the normal regions of images, and instead it could introduce new anomalies. To address these challenges, unlike DRAEM, we not only input the original and reconstructed images but also incorporate the feature layer information obtained during the reconstruction process. The hierarchical information input provides additional sources of information for the discriminative sub-network, thus mitigating the network’s tendency to solely rely on clear boundaries to locate anomaly regions. Moreover, it offers the network prior information about regions where the reconstruction may fail. Specifically, the input feature layer information consists of the feature maps output from the third, fifth, and eighth layers of the reconstructive sub-network. These feature maps undergo convolutional operations and are resized to the size of the original image, with the number of channels reduced to one. After passing through normalization and activation layers, these feature maps are concatenated with the original image and the reconstructed image, thereby forming the hierarchical information input.

The CNN-based U-Net structure struggles to strike a balance between classification accuracy and localization precision. When the receptive field is selected to be relatively large, the downsampling multiplier of the subsequent pooling layer will increase, leading to a decrease in localization accuracy. However, when the receptive field is relatively small, we will observe a decrease in classification accuracy [23]. Therefore, unlike DRAEM, we adopt both Swin transformer and UperNet. Swin transformer can effectively capture both global and local information, and it shows strong feature extraction and semantic learning capabilities in various tasks. Meanwhile, UperNet efficiently fuses multi-scale feature maps and progressively performs image upsampling, thus enabling the generation of high-quality prediction maps. By combining the strengths of both, our discriminative sub-network’s ability to detect and localize anomalies in images is further improved.

Due to the low percentage of defective regions across a map and the fact that many defects are very similar to the background of the picture, our discriminative sub-network uses focal loss [24] to create constraints. Combined with the reconstructive sub-network, the total loss of training is:

(5) $$L\left(I_{r},I,Map,GT\right)=L_{rec}\left(I_{r},I\right)+L_{seg}\left(Map,GT\right).$$

where *GT* is the ground truth.

## 5. Experiments

In this section, we conduct evaluation experiments on the MVTec AD and GTanoIC datasets to assess the proposed method’s performance. We also compare it with other reconstruction-based anomaly detection models. In addition, we validate the effectiveness of each of its components through ablation experiments.

### 5.1. Datasets, Metrics, and Implementation Details

#### 5.1.1. Datasets

In addition to the GTanoIC chip dataset, we also perform evaluation experiments on the widely used public dataset MVTec AD. This dataset contains a total of 5354 high-resolution images of 15 objects including industrial parts, daily products, and finished fabrics; it also provides pixel-level annotations for anomalous images.

#### 5.1.2. Evaluation Metrics

As in mainstream anomaly network models [15,16,17,25], we leverage image-level and pixel-level AUROC for performance evaluation.

#### 5.1.3. Implementation Details

We set the number of training epochs to 700 and the batch size to 4, and then we performed experiments in an A40 graphics environment. The proposed intermediate feature layer fusion was spliced in a sequential manner, where the patch_size of Swin transformer was set to 4, image_channel was set to 15, embed_dim was set to 128, depth was set to (2, 2, 42, 4), and num_headps was set to (4, 8, 16, 32). We followed the MVTec AD dataset evaluation criteria and used [256, 256] as the H and W of the chip dataset images for these experiments. Note that the chip dataset was constructed with real scenarios; differences in where and how images were collected resulted in chips occupying different proportions of the images. Therefore, CropAndPad was used for pre-processing to make the chip size consistent across images. In addition to this, in the section where the images were augmented, we randomly selected four out of the ten kinds of data enhancement to act on the simulated defect.

### 5.2. Comparison with Existing Methods

On the MVTec AD dataset, our proposed model was compared with other recently developed unsupervised anomaly detection models based on image reconstruction: RIAD, CutPaste, InTra, EdgRec, OCRGAN, and DRAEM.

The evaluation results for anomaly detection are shown in Table 3. Among the 15 different sub-datasets (containing different classes of objects), our model outperforms the other models in 10, with the most significant improvement observed in detecting images in texture categories.

The evaluation results for anomaly localization are shown in Table 4. Among the 15 different sub-datasets, our model outperforms the other models in 5, with the most significant improvement observed in detecting images in object categories.

For experiments on the GTanoIC dataset, note that we excluded CutPaste and InTra from comparisons since they do not provide a source code. The results are shown in Table 5 and Table 6.

From the above experiments, it can be seen that the performance of our proposed anomaly detection model is significantly better than other similar methods. Compared with DRAEM, the image-level AUROC is improved by 11.6% on average, and the pixel-level AUROC is improved by 6% on average.

**Figure 7:**
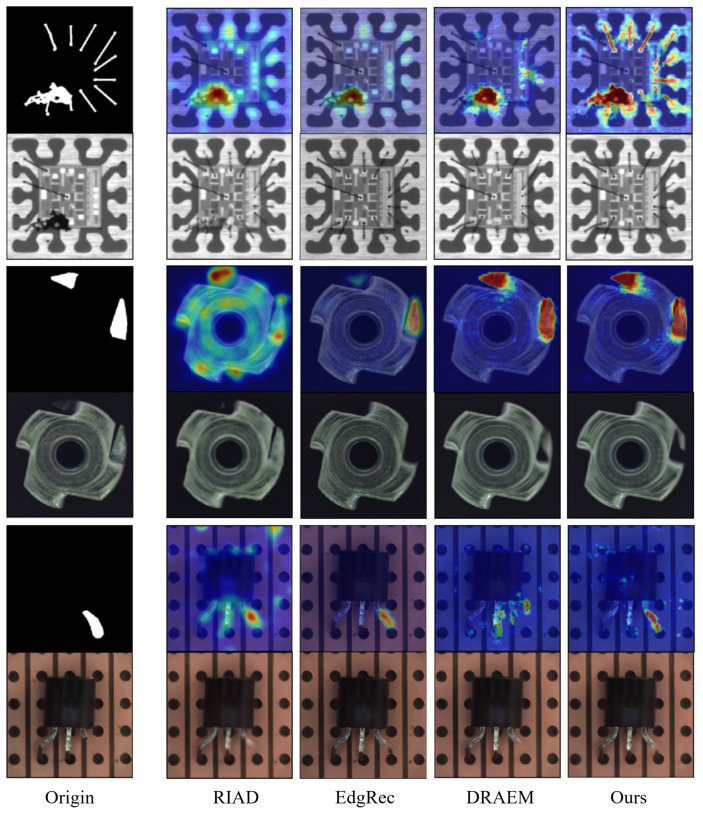
Visualization of anomaly localization results of various methods on the GTanoIC and MVTec AD datasets. Input images with ground truth masks (**left**), reconstructed images and predicted anomaly maps of various methods (**right**) are provided.

Figure 7 shows the results of anomaly localization performed by various methods on the test images of GTanoIC and MVTec AD datasets.

## 6. Ablation Study

We conducted an ablation study to verify the effect of using each of the three components of our proposed algorithm: progressive reconstruction, Swin transformer and UperNet, and hierarchical feature fusion.

To further improve the quality of a reconstructed image without increasing the network’s generalizability, we proposed a progressive reconstruction approach. We conducted experiments with one to five iterations, as shown in Table 7. On both datasets, we observed an improvement in AUROC after the second reconstruction, but further reconstructions did not lead to continued improvement. Instead, they resulted in a decline.

**Figure 8:**
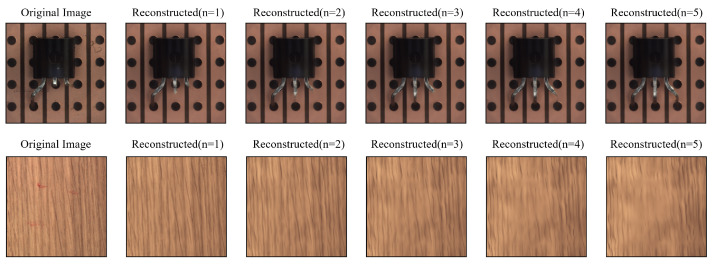
As the number of reconstructions increases (n > 2), the structure-type patterns are gradually fully repaired, yet the texture-type patterns tend to become blurred.

Through further analysis, we found that for structural patterns, better reconstruction can generally be achieved. For example, in Figure 8, the transistor pattern is gradually repaired and becomes complete with an increase in the number of reconstructions. However, for textured patterns, the results are not satisfactory, and the image quality decreases as the number of reconstructions increases. As a result, for objects with different types of defects, we can choose different numbers of iterations to improve the quality of reconstructed images.

Compared with U-Net, combining Swin transformer and UperNet allows us to focus on both local and global information, thus ensuring classification accuracy while improving localization accuracy. Meanwhile, in the training phase, the inclusion of feature layer information prevents the discriminative sub-network from outputting the difference between reconstructed and alienated maps based only on clear boundaries of simulated defects. In the detection phase, high-dimensional features of the reconstruction process provide the network with a wealth of information to improve robustness. Ablation results on these two components can be seen in Table 8.

## 7. Conclusions

In this paper, we propose a progressive reconstruction and hierarchical feature-fusion-based method for anomaly detection and localization that can be used for defect detection in various products such as chips. Our method outperforms other reconstruction-based methods on the challenging MVTec AD dataset. In addition, to the best of our knowledge, we have constructed the largest real chip surface defect dataset (GTanoIC); experiments on this dataset reveal that the proposed method significantly outperforms similar algorithms.

In future work, we will explore the use of more capable networks such as diffusion models [26] to better reconstruct defective images and make the progressive reconstruction remain convergent. At the same time, we will try to limit the network’s resilience to anomalous areas through supervised learning.

## Figures and Tables

**Table 1:** Statistical information on flip chips.

Dataset	Type	Pollution	Crack	Broken	No Die	Foreign Substance	Non- Defective	Resolution
$D_{A}$	instance-level	31	3	19	/	7	250	350*400
	image-level	60 defective images in total						
$D_{B}$	instance-level	40	/	9	7	4	250	340*400
	image-level	60 defective images in total						
$D_{C}$	instance-level	37	2	16	/	5	250	260*285
	image-level	60 defective images in total						
$D_{D}$	instance-level	52	/	12	/	6	250	250*200
	image-level	70 defective images in total						
$D_{E}$	instance-level	63	/	/	6	/	250	260*270
	image-level	69 defective images in total						

**Table 2:** Statistical information on chips with solder wires.

Dataset	Type	Pollution	No Wires	No-Die	Wire Residue	Non- Defective	Resolution
$D_{F}$	instance-level	75	1	5	6	250	340*340
	image-level	87 defective images in total					
$D_{G}$	instance-level	52	1	/	11	250	330*330
	image-level	64 defective images in total					

**Table 3:** Anomaly detection results on the MVTec AD dataset (image-level AUROC).

Category		RIAD	CutPaste	InTra	EdgRec	DRAEM	OCRGAN	Ours
Texture	Carpet	84.2	93.1	98.8	97.4	97.0	**99.4**	99.0
	Grid	99.6	99.9	**100.0**	99.7	99.9	99.6	99.9
	Leather	**100.0**	**100.0**	**100.0**	**100.0**	**100.0**	97.1	**100.0**
	Tile	98.7	93.4	98.2	**100.0**	99.6	95.5	**100.0**
	Wood	93.0	98.6	97.5	94.0	99.1	95.7	**99.6**
Object	Bottle	99.9	98.3	**100.0**	**100.0**	99.2	99.6	99.8
	Capsule	88.4	96.2	86.5	95.5	**98.5**	96.2	98.0
	Pill	83.8	92.4	90.2	**99.0**	98.9	98.3	98.2
	Transistor	90.9	95.5	95.8	99.8	93.1	98.3	**97.4**
	Zipper	98.1	99.4	99.4	98.3	**100.0**	99.0	**100.0**
	Cable	81.9	80.6	70.0	97.9	91.8	99.1	**96.9**
	Hazelnut	83.3	97.3	95.7	98.4	**100.0**	98.5	99.9
	Matal nut	88.5	99.3	96.9	97.3	98.7	99.5	**99.6**
	Screw	84.5	86.3	95.7	89.9	93.9	**100.0**	98.7
	Toothbrush	**100.0**	98.3	**100.0**	**100.0**	**100.0**	98.7	**100.0**
Average${}_{tex}$		95.1	97.0	98.9	98.2	99.1	97.5	**99.7**
Average${}_{obj}$		89.9	94.4	93.0	97.6	97.4	98.7	**98.9**
Average		91.7	95.2	95.0	97.8	98.0	98.3	**99.1**

**Table 4:** Anomaly localization results on the MVTec AD dataset (pixel-level AUROC).

Category		RIAD	CutPaste	InTra	EdgRec	DRAEM	OCRGAN	Ours
Texture	Carpet	96.3	98.3	99.2	**99.4**	95.5	-	94.0
	Grid	98.8	97.5	98.8	99.2	**99.7**	-	99.6
	Leather	99.4	99.5	99.5	99.7	98.6	-	**99.8**
	Tile	89.1	90.5	94.4	98.6	**99.2**	-	98.8
	Wood	85.8	95.5	88.7	91.4	**96.4**	-	95.5
Object	Bottle	98.4	97.6	97.1	98.3	**99.1**	-	99.0
	Capsule	92.8	97.4	97.7	95.2	94.3	-	**97.9**
	Pill	95.7	95.7	98.3	98.7	97.6	-	**98.5**
	Transistor	87.7	93.0	**96.1**	94.3	90.9	-	94.3
	Zipper	97.8	**99.3**	99.2	98.7	98.8	-	98.8
	Cable	84.2	90.0	91.0	97.7	94.7	-	**96.5**
	Hazelnut	96.1	97.3	98.3	99.4	**99.7**	-	99.5
	Matal nut	92.5	93.1	93.3	98.0	**99.5**	-	98.4
	Screw	98.8	96.7	**99.5**	97.7	97.6	-	99.3
	Toothbrush	98.9	98.1	98.9	99.2	98.1	-	**99.5**
Average${}_{tex}$		93.9	96.3	96.1	**97.7**	97.6	-	97.5
Average${}_{obj}$		94.3	95.8	96.9	97.7	97.0	-	**98.2**
Average		94.2	96.0	96.6	97.7	97.3	-	**98.0**

**Table 5:** Anomaly detection results on the GTanoIC dataset (image-level AUROC).

Category	OCRGAN	DRAEM	RIAD	EdgRec	Ours
$D_{A}$	77.0	99.6	95.4	**100.0**	99.6
$D_{B}$	60.6	**100.0**	98.8	100.0	99.7
$D_{C}$	91.3	97.8	55.5	**99.9**	99.3
$D_{D}$	76.1	94.3	96.1	**99.4**	98.1
$D_{E}$	60.1	76.3	80.1	85.8	**92.6**
$D_{F}$	98.7	94.6	95.7	89.3	**95.8**
$D_{G}$	43.1	38.4	80.3	91.5	**97.2**
Average	69.7	85.9	86.0	95.1	**97.5**

**Table 6:** Anomaly detection results on the GTanoIC dataset (pixel-level AUROC).

Category	OCRGAN	DRAEM	RIAD	EdgRec	Ours
$D_{A}$	-	93.6	88.7	96.5	**99.0**
$D_{B}$	-	99.4	97.6	98.9	**99.6**
$D_{C}$	-	93.6	79.6	95.1	**98.8**
$D_{D}$	-	93.6	91.4	96.9	**97.7**
$D_{E}$	-	93.9	96.6	94.8	**98.0**
$D_{F}$	-	85.2	91.0	87.8	**96.4**
$D_{G}$	-	86.7	93.1	96.4	**98.7**
Average	-	92.3	91.1	95.2	**98.3**

**Table 7:** Ablation results on the MVTec and GTanoIC datasets (progressive reconstruction component).

Dataset	Type	n = 1	n = 2	n = 3	n = 4	n = 5
MVTec AD	Image-level	98.9	**99.1**	99.1	99.0	99.0
	Pixel-level	97.6	**98.0**	97.7	97.5	97.2
GTanoIC	Image-level	97.5	**97.5**	97.5	97.5	97.5
	Pixel-level	98.1	**98.3**	98.3	98.2	98.0

**Table 8:** Ablation results on the MVTec and GTanoIC datasets (Swin transformer and UperNet component plus the hierarchical feature fusion component).

Dataset	Swin. and	Hierarchical	Image-Level	Pixel-Level
	**UperNet**	**Feature Fusion**	**AUROC**	**AUROC**
	-	-	98.0	97.3
MVTec AD	✔	-	98.2	97.4
	✔	✔	**99.1**	**98.0**
	-	-	94.8	97.3
GTanoIC	✔	-	96.4	98.0
	✔	✔	**97.5**	**98.3**

## Data Availability

The GTanoIC dataset is now available at https://github.com/HiHiAllen/GTanoIC-Dataset-for-AD, accessed on 1 September 2023 (GTanoIC-Dataset-for-AD).
